# Supplementary material for: Robot-assisted, source-camera-coupled multi-view broadband imagers for ubiquitous sensing platform
Source: Nat Commun. 2021 May 21;12:3009. doi: 10.1038/s41467-021-23089-w (PMC8139987; doi:10.1038/s41467-021-23089-w)
Supplement: Supplementary file 1 — Supplementary Information [file 41467_2021_23089_MOESM1_ESM.pdf]

Supplementary Information of

Robot-assisted, source-camera-coupled multi-view broadband imagers for ubiquitous sensing platform

Kou Li[1], Ryoichi Yuasa[1], Ryogo Utaki[1], Meiling Sun[1], Yu Tokumoto[1], Daichi Suzuki[2], Yukio Kawano[1,*]

[1]Laboratory for Future Interdisciplinary Research of Science and Technology, Tokyo Institute of Technology, 2-12-1 Ookayama, Meguro-ku, Tokyo 152-8552, Japan
[2]Center for Emergent Matter Science, RIKEN, 2-1 Hirosawa, Wako-shi, Saitama 351-0198, Japan

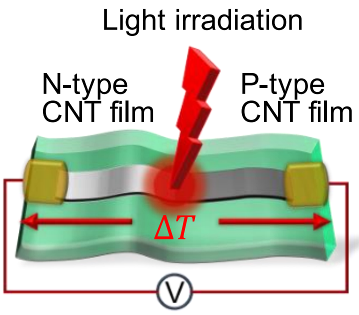

$$\Delta V = (S_{\mathrm{P-CNT}} - S_{\mathrm{N-CNT}}) \times \Delta T$$

ΔV : Photo-induced PTE voltage response

S : Seebeck coefficient ($S_{\mathrm{P\text{-}CNT}}$: 45 μV/K, $S_{\mathrm{N\text{-}CNT}}$: −40 μV/K)

ΔT : Photo-induced thermal gradient

Channel size : 1 mm (W) × 5 mm (L) × 1 μm (T)

Supplementary Figure 1.
Photo-thermoelectric (PTE) effect with the carbon nanotube (CNT) film sensor.
W: width, *L*: length, *T*: thickness.

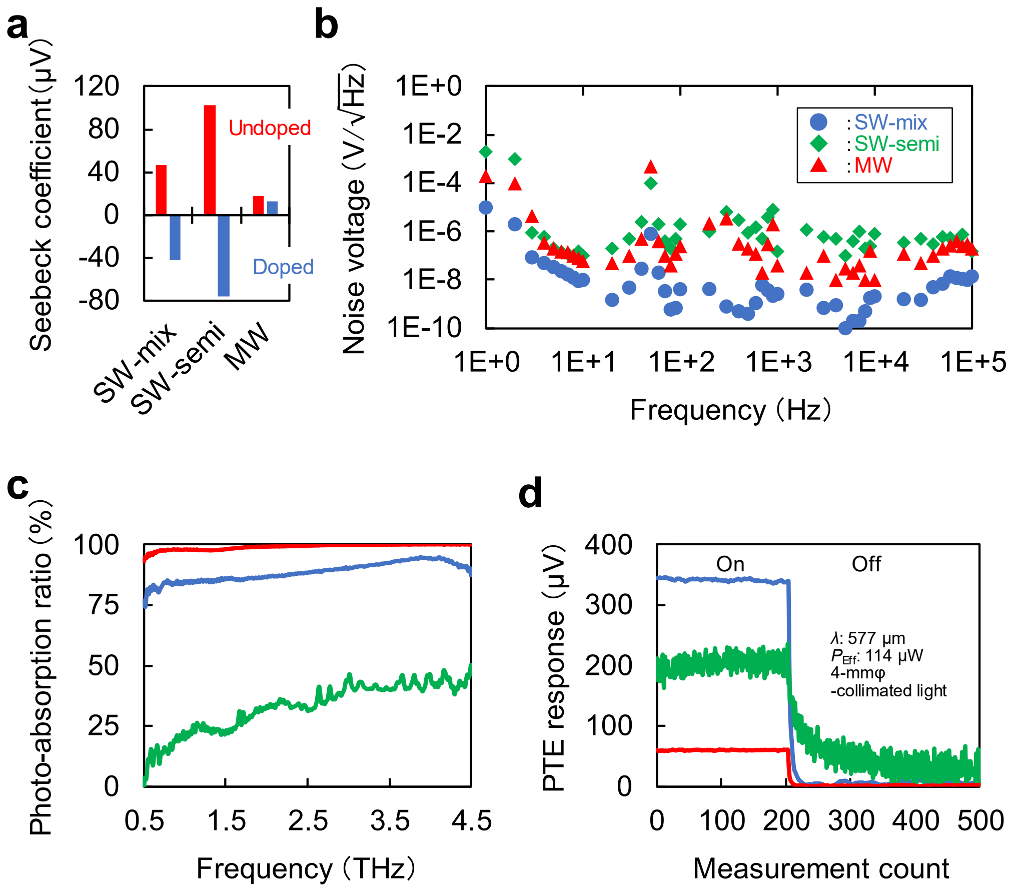

Supplementary Figure 2.

Comparison of photo-thermoelectric (PTE) properties of semiconducting and metallic mixed-type single-walled carbon nanotube (SWCNT) film, semiconducting-separated SWCNT film, and multi-walled CNT (MWCNT) film with PN junctions.

In addition to the employed semiconducting and metallic mixed-type SWCNT film, there are other potential CNT-related material candidates for the ubiquitous photo-imager channel, including the semiconducting-separated SWCNT film (for its efficient thermoelectric conversion), and the MWCNT film (for its high photo-absorption characteristic). **a,** Seebeck coefficients with and without liquid coating N-type chemical doping (0.4 mol/L). A semiconducting-separated undoped SWCNT solution (RedSemicon Ink, Meijo Nano Carbon Co., LTD.) and a 1-mm-thick MWCNT paper (S01-01, AS ONE Co.) were respectively employed. Here, each channel was formed under the following conditions: a thickness of 1 μm, a length of 4 mm, and a width of 0.5 mm. Together with the experimental comparisons of **b,** the noise voltage, and **c,** the photo-absorbance ratio in the THz frequency region, the semiconducting and metallic mixed-type SWCNT film channel exhibited **d,** superior terahertz (THz) photo-detection performance (λ = 577 μm) compared to the semiconducting-separated SWCNT film channel and MWCNT film channel. Here, the half side of each channel was applied with the N-type chemical doping for (**b**, **d**), and the electrical resistances are as follows: 191 Ω for the semiconducting and metallic mixed-type SWCNT film channel, 163 kΩ for the semiconducting-separated SWCNT film channel, and 140 Ω for the MWCNT film channel. Although the semiconducting-separated SWCNT film shows the highest Seebeck coefficient among the candidates, its lowest photo-absorption characteristic suppresses its photo-response. On the other hand, although the MWCNT film shows the highest photo-absorption characteristic, its lowest Seebeck coefficient also suppresses its photo-response. Therefore,

the semiconducting and metallic mixed-type SWCNT film is most suitable for the flexible broadband photo-imager channel among the candidate materials.

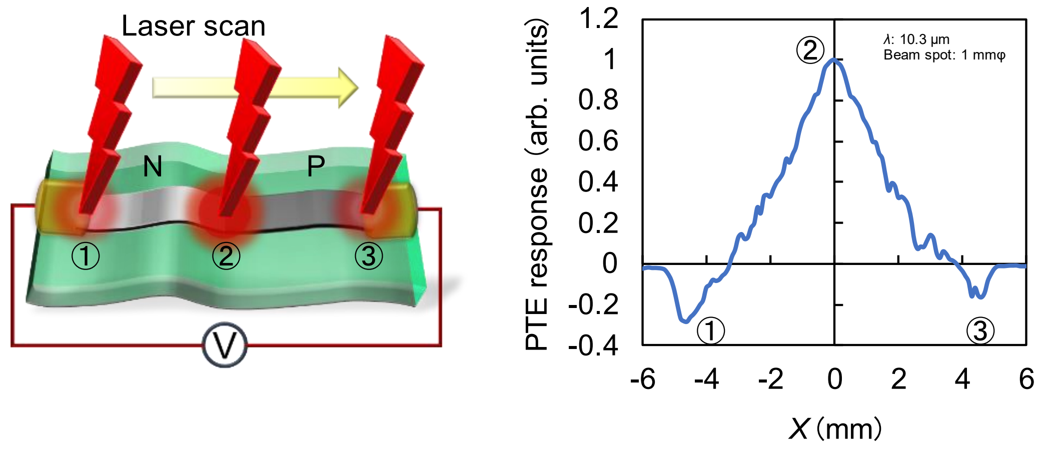

Supplementary Figure 3.

Comparison of photo-thermoelectric (PTE) responses obtained among the electrode-N type carbon nanotube (CNT) film channel junction, PN junction of the CNT film channel, and P type CNT film channel-electrode junction.

The graph indicates that higher intensity PTE response can be obtained on the PN junction of the CNT film channel compared to those acquired on the CNT film channel-electrode interfaces. The PTE effect at the different materials interfaces enables us to obtain the voltage response by multiplying the effective Seebeck coefficient between the materials employed and photo-induced thermal gradient, as mentioned in Fundamental device design for a CNT-film flexible broadband PTE photo-imager of the Introduction section. Here, PTE responses of each photo-detection interface illustrated in the figure can be described as below:

① : $$\Delta V = S_{\mathrm{Eff}} \times \Delta T = (S_{\mathrm{N-CNT\ film}} - S_{\mathrm{Electrode}}) \times \Delta T \quad (1)$$

② : $$\Delta V = S_{\mathrm{Eff}} \times \Delta T = (S_{\mathrm{P-CNT\ film}} - S_{\mathrm{N-CNT\ film}}) \times \Delta T \quad (2)$$

③ : $$\Delta V = S_{\mathrm{Eff}} \times \Delta T = (S_{\mathrm{Electrode}} - S_{\mathrm{P-CNT\ film}}) \times \Delta T \quad (3)$$

We note that each Seebeck coefficient was measured as $S_{\mathrm{P-CNT\ film}}$: 47 μV/K, $S_{\mathrm{N-CNT\ film}}$: −42 μV/K, $S_{\mathrm{Electrode}}$: 1.5 μV/K. Equations (1)–(3) also infer that forming a PN junction of the CNT film results in an enhancement of the effective Seebeck coefficient, in which the absolute values of $S_{\mathrm{P-CNT\ film}}$ and $S_{\mathrm{N-CNT\ film}}$ are added together. On the other hand, the absolute values of each Seebeck coefficient of the CNT film-electrode interface are suppressed and smaller than that of the CNT film PN junction. Thus, forming the PN junction of the CNT film channel is essential for PTE response enhancement, where the effective Seebeck coefficient of the photo-detection interface can be maximised, and was employed in the proposed device structure.

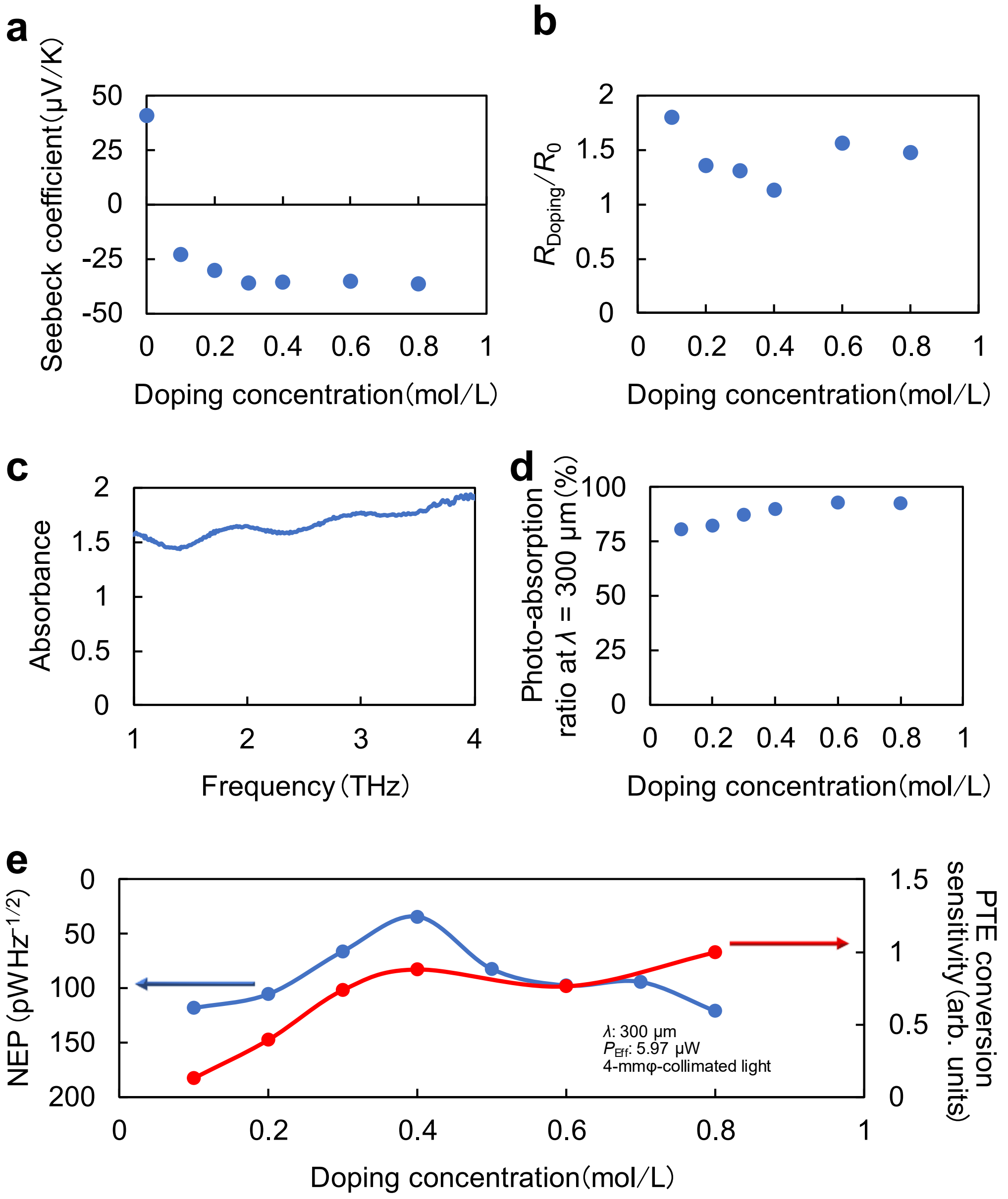

Supplementary Figure 4.

Change in photo-thermoelectric (PTE) parameters of carbon nanotube (CNT) film sensor in response to the concentration of the N-type chemical-carrier dopant.

a, Mapping of the CNT film Seebeck coefficient in response to the N-type doping concentration on the channel. **b,** Mapping of the photo-imager electrical resistance in response to the N-type doping concentration on the CNT film channel. **c,** The absorbance spectrum of the chemically N-type-doped CNT film (0.1 mol/L concentration) in the terahertz (THz) frequency region. The measurement was performed by utilising the THz time domain spectroscopy (THz-TDS). **d,** Mapping of the CNT film photo-absorption ratio in the THz (λ = 300 μm) frequency region in response to the N-type doping concentration. **e,** Change in the noise equivalent power (NEP) value and the PTE conversion sensitivity vs. N-type doping concentration. The presented PTE conversion tuning improved the NEP (120 pWHz$^{-1/2}$ → 30 pWHz$^{-1/2}$, in the THz frequency region) and the minimum NEP value of 8.57 pWHz$^{-1/2}$ was observed under the condition of the uncooled non-vacuum far-infrared (FIR: λ = 10.3 μm) photo-detection with 0.4-mol/L-concentration N-type chemical-carrier doping on the CNT film photo-imager.

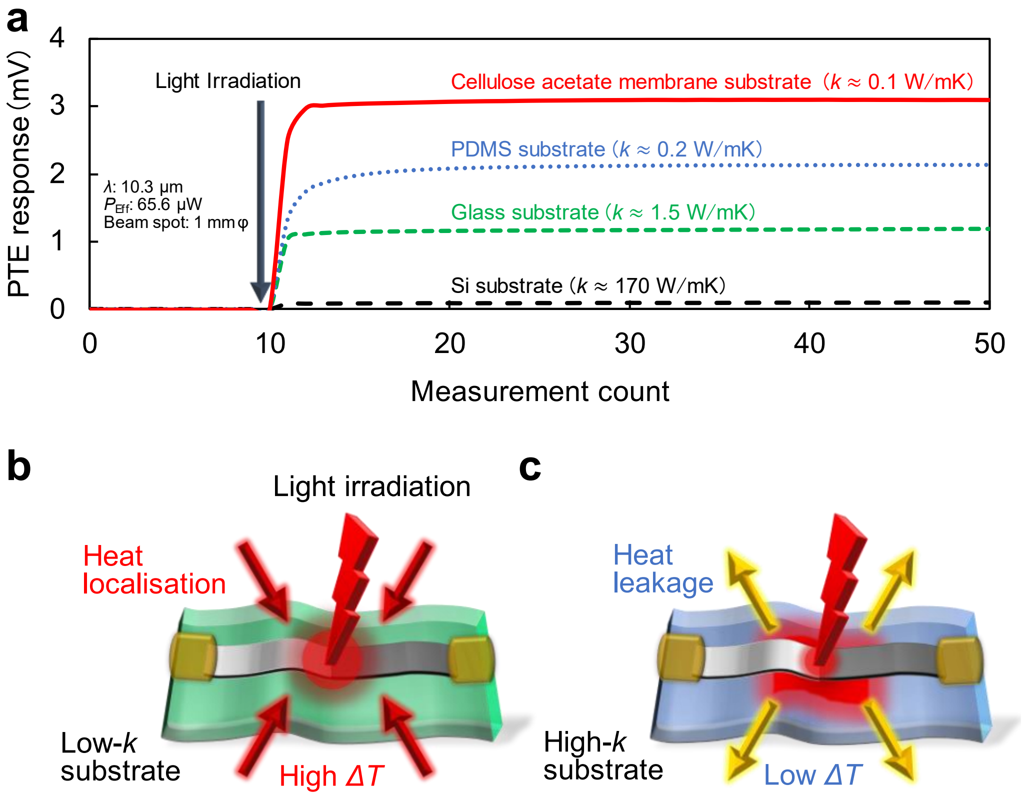

Supplementary Figure 5.

ΔT low-loss thermal device design of a carbon nanotube (CNT) film photo-thermoelectric (PTE) sensor. **a,** Comparison of photo-induced PTE responses of CNT film photo-imager with different types of supporting substrate among flexible (cellulose acetate membrane substrate, Polydimethylsiloxane (PDMS) substrate) and rigid (glass substrate, Silicon (Si) substrate) materials in the far-infrared (FIR: $\lambda = 10.3$ μm) frequency region. The results indicate that the photo-induced PTE response intensity is highly dependent on the thermal conductivity k of supporting substrates. In particular, there was up to 30 times difference between the photo-induced PTE response intensity acquired by the CNT film photo-imager with the cellulose acetate membrane substrate and that with the Si substrate. **b,** Schematic of the photo-detection using a CNT film photo-imager with a lower thermal conductivity supporting substrate. Here, the use of substrates with lower thermal conductivity functions as a simple heat localization device design in terms of the photo-induced heating in the CNT film channel, avoiding the heat leakage from the CNT film channel to the supporting substrate. Therefore, higher photo-induced PTE responses obtained by CNT film photo-imagers with lower thermal conductivity supporting substrates can be attributed to both the present simple heat localisation structure and the fact that the photo-induced PTE voltage signals are proportional to the photo-induced thermal gradient ΔT from Eq. 1. **c,** Schematic of the photo-detection using a CNT film photo-imager with a higher thermal conductivity supporting substrate. Here, the use of substrates with higher thermal conductivity causes the leakage of the photo-induced heating in the CNT film channel to the supporting substrate, which is thought to trigger ΔT degradation and consequential lower photo-induced PTE responses. In light of the above discussion, we employed the cellulose acetate membrane supporting substrate from the viewpoint of its mechanical flexibility and lower thermal conductivity among other materials assessed in this study.

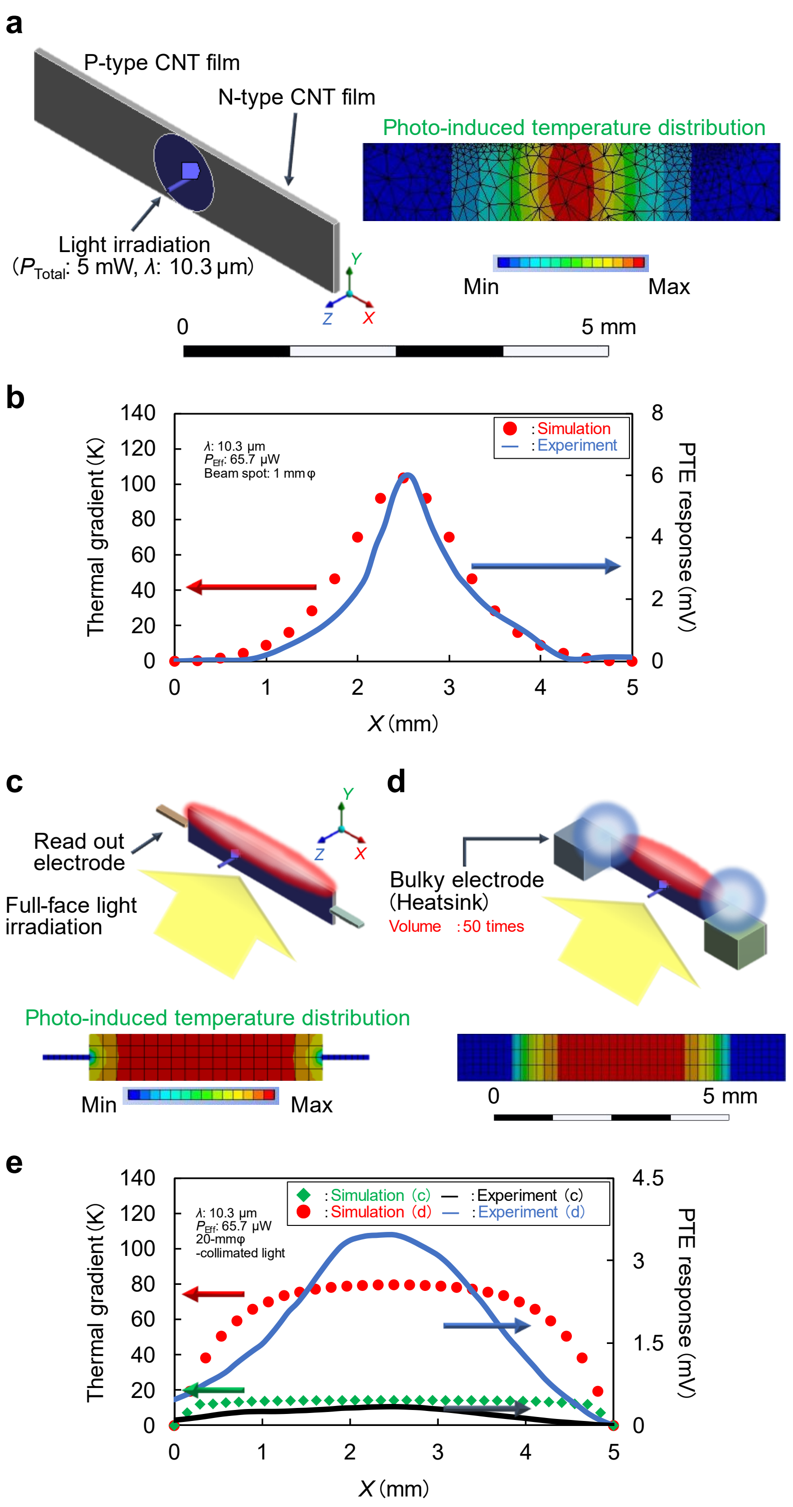

Supplementary Figure 6.

Thermal device design for the full-face light irradiation.

The figure shows the spatial resolution and the dependence of irradiated light beam-shape on photo-detection responses of the carbon nanotube (CNT) film photo-thermoelectric (PTE) sensor from the viewpoint of the photo-induced temperature distribution in the channel. **a,** Analysis model of the steady-state thermal distribution under the condition of light irradiation focused onto the PN junction of the CNT film photo-imager. Steady-state thermal simulation of the Methods section describes the corresponding detailed conditions. **b,** Mapping of the experimental photo-induced PTE response and the simulated thermal gradient of the CNT film photo-imager with the focused far-infrared (FIR: λ = 10.3 μm) light irradiation. The photo-induced PTE response mapping was obtained by scanning the irradiated light along the channel length direction with a

digital stepping motor. The result indicates that the focused light irradiation brought a sharp single peak of the photo-induced PTE response mapping and thermal gradient mapping, which are thought to be particularly important for the spatial resolution and the image quality[1,2]. **c,** Steady-state thermal distribution simulation of the CNT film photo-imager under the full-face light irradiation condition. In contrast to the simulated result for the focused light irradiation, the reduction of photo-induced thermal gradient ΔT due to the temperature rise throughout the channel area was observed. This means that the operation of the CNT film photo-imager under the full-face light irradiation causes photo-detection performance degradation in terms of not only the lower photo-detection response, but also the lower spatial resolution due to an increment of the half-width at half maximum (HWHM) values[3]. **d,** Steady-state thermal distribution simulation of the CNT film photo-imager with a simple heatsink structure under the full-face light irradiation. The proposed structure consists of bulky stretchable readout electrodes at the ends of the channel, which was designed to be 50 times larger in volume (5 times width,10 times thickness) compared to those shown in (**c**). The stretchable readout electrode itself is composed of a silver nanowire paste, thereby the formation of bulky readout electrodes, which shows a higher thermal conductivity k than that of the CNT film ($k_{\text{CNT film}}$: $k_{\text{Silver nanowire paste}}$ = 1:19)[3,4] relaxes the temperature rise across the channel area by facilitating local heat leakages to electrodes. **e,** Mapping of the experimental photo-induced PTE responses and the simulated thermal gradients of the CNT film photo-imagers with and without the proposed simple heatsink structure under the full-face FIR light irradiation. Five-fold enhancement in the photo-induced PTE response and ten-fold improvement in the HWHM value (from 2.35 to 1.2 mm) with the simple heatsink structure were observed in the obtained results. While the module driving under the full-face light irradiation is potentially demanded toward the operation-environment-independent ubiquitous sensing, the presented thermal device design facilitates the prevention of photo-detection performance degradation, and the proposed CNT film photo-imager is operable regardless of the irradiated light beam-shape.

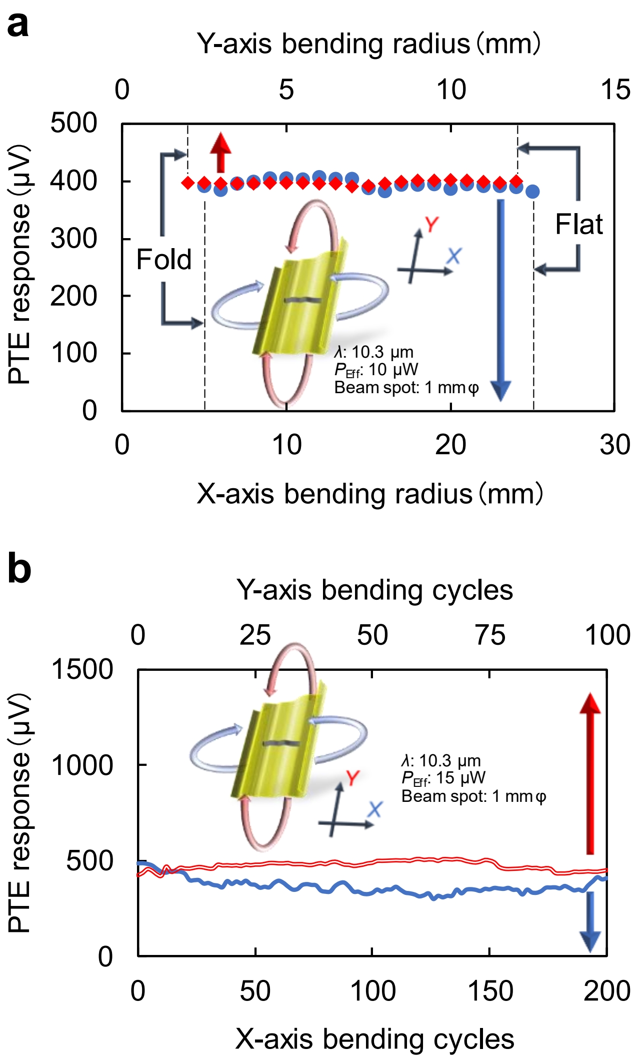

Supplementary Figure 7.

Stability of the photo-thermoelectric (PTE) conversion against folding of the whole carbon nanotube (CNT) film flexible photo-imager.

We investigated the optical stability of the CNT film in terms of mechanical flexibility. The following measurements were performed by monitoring the photo-induced PTE conversion in the far-infrared (FIR: λ = 10.3 μm) frequency region, while the CNT film photo-imager was continuously bent by utilising an optical jig. **a,** Mapping of photo-induced PTE voltage signals in response to the bending radius in both channel length and width directions. Uniform behaviour of the photo-induced PTE conversion was observed even under the full-bending condition, and the result highlights the optical stability of the photo-imager against the omni-directional whole sheet device folding. **b,** Mapping of photo-induced PTE voltage signals in response to the number of repetitive bending cycles in both channel length and width directions. Uniform behaviour of the photo-induced PTE conversion was observed up to 200 times bending, and the result further represents the optical stability of the photo-imager against the hundreds of repetitive omni-directional whole sheet device folding.

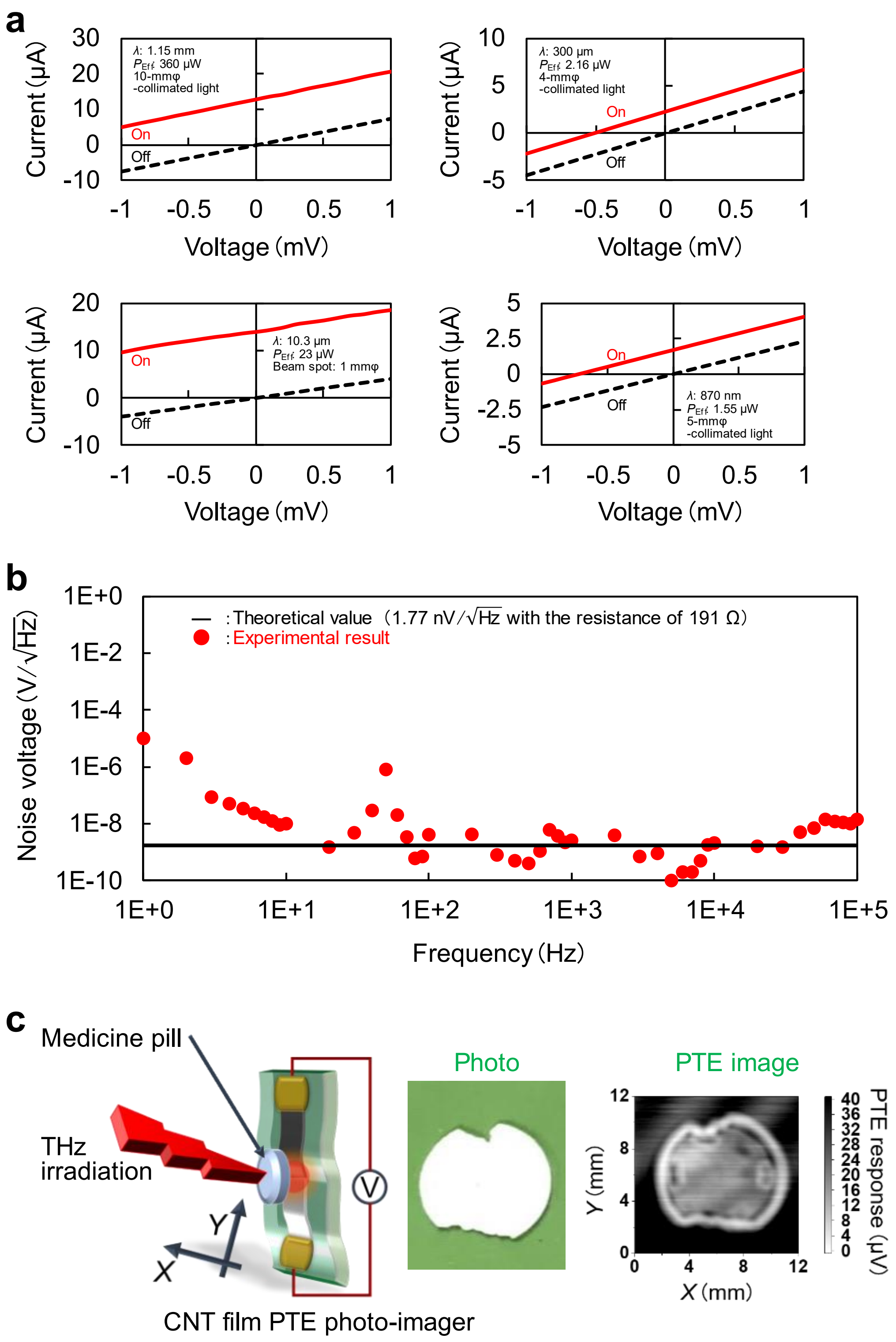

Supplementary Figure 8.

Fundamental performance of the carbon nanotube (CNT) film photo-thermoelectric (PTE) broadband imager.

a, Current-voltage characteristics with and without the light irradiation in the broadband frequency regions ranging from the sub-terahertz (sub-THz: 260 GHz, λ = 1.15 mm) to the THz (1 THz, λ = 300 μm) to the far-infrared (FIR: 29 THz, λ = 10.3 μm) to the near-infrared (NIR: 345 THz, λ = 870 μm) bands. The results verified not only the broadband photo-detection of the CNT film but also the zero-bias voltage operation. **b,** Noise voltage mapping of the CNT film photo-imager. Owing to the zero-bias voltage operation, the noise voltage values were suppressed down to the theoretical thermal noise value. This theoretical value was

calculated by the function expressed in NEP evaluation of the Methods section. The peak value at 50 Hz originates from the power source of the measurement system (not from the CNT film photo-imager). **c,** Demonstration of the transmissive XY-scan photo-monitoring with a single pixel of the photo-imager in the THz frequency region. The transmissive image collectively captures the photo-absorption at the centre of the target medicine pill and the scattering at the pill edge. Stepping motor scanning of the Methods describes the corresponding detailed conditions.

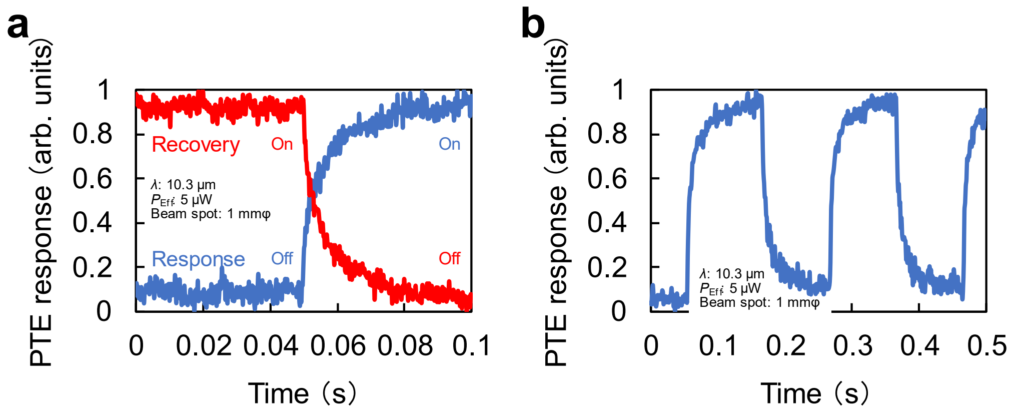

Supplementary Figure 9.

Operation speed of carbon nanotube (CNT) film photo-thermoelectric (PTE) sensor.

a, Transient photo-response of the device in the far-infrared (FIR) frequency region (λ = 10.3 μm). The response and recovery time constants can be calculated to be 5 ms and 5.2 ms, respectively, from the results.

b, Photo-response mapping of the proposed device with on-off switching of the FIR irradiation.

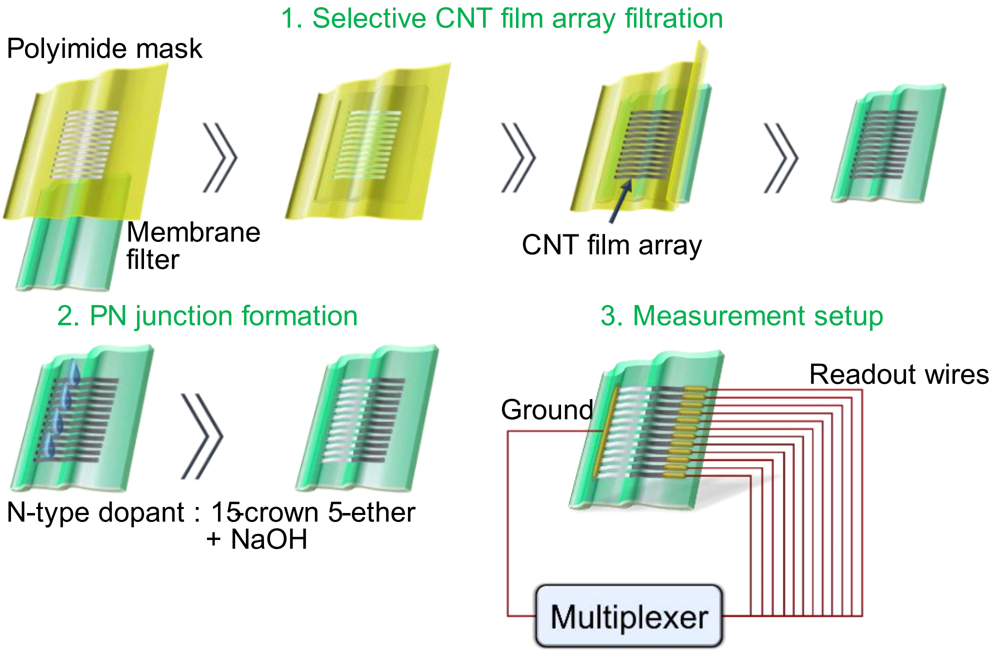

Supplementary Figure 10.

Schematic fabrication flow of the multi-pixel carbon nanotube (CNT) film photo-thermoelectric (PTE) image sensor array sheet.

Selective suction CNT film patterning, Chemical carrier doping, Stretchable electrode of the Methods section describe the corresponding detailed conditions.

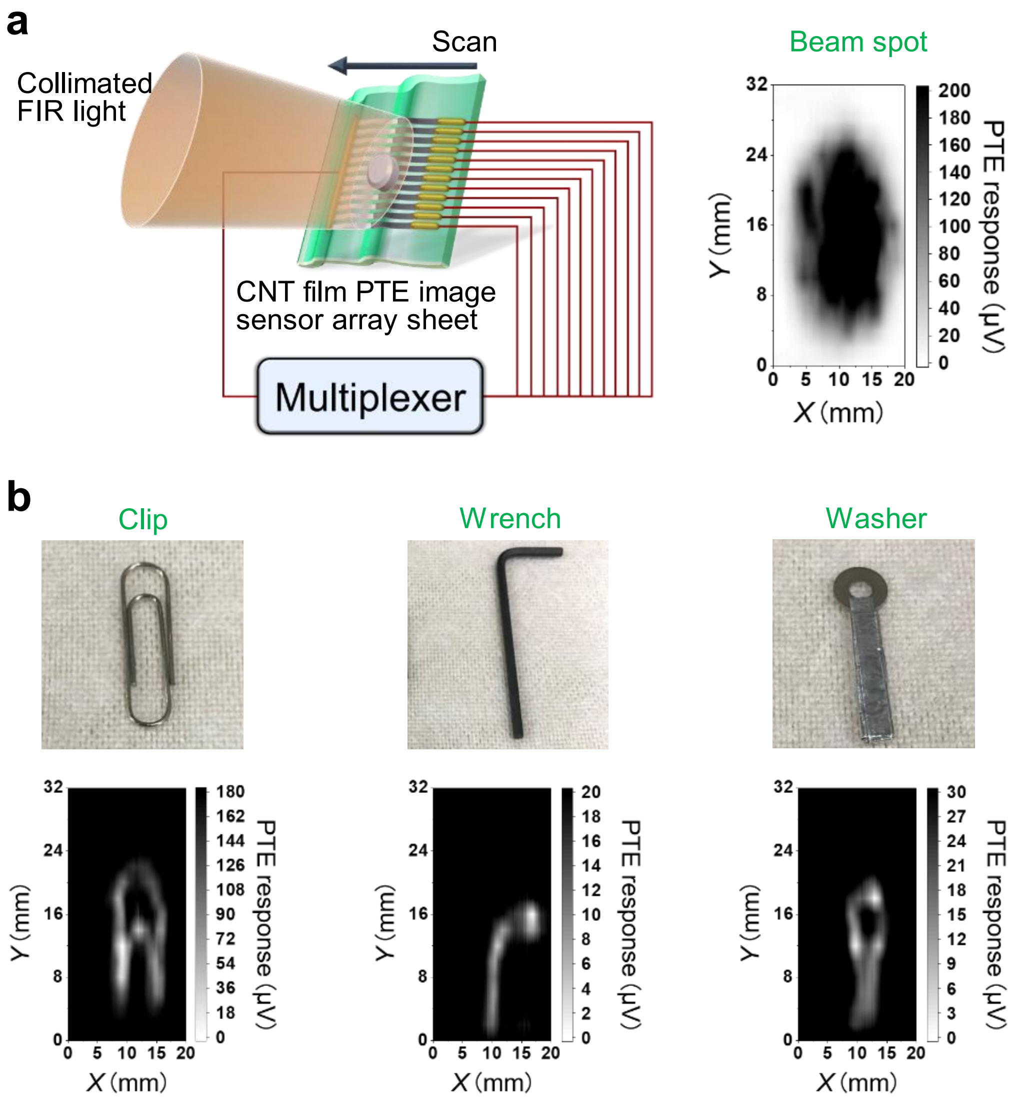

Supplementary Figure 11.

Fundamental photo-imaging performance of the multi-pixel carbon nanotube (CNT) film photo-thermoelectric (PTE) sensor array sheet.

a, Measurement setup for the transmissive photo-imaging in the far-infrared (FIR: λ = 10.3 µm) frequency region. Here, we irradiated 20-mmφ collimated FIR light, and Multi-pixel photo-induced PTE voltage signal readout of the Methods section describes the corresponding detailed conditions. **b,** Transmissive one-dimensional array scan photo-imaging of the metallic clip, wrench, and washer. The resulting images indicate that the utilisation of the multi-pixel CNT film PTE sensor sheet can function as a photo-monitoring array scanner.

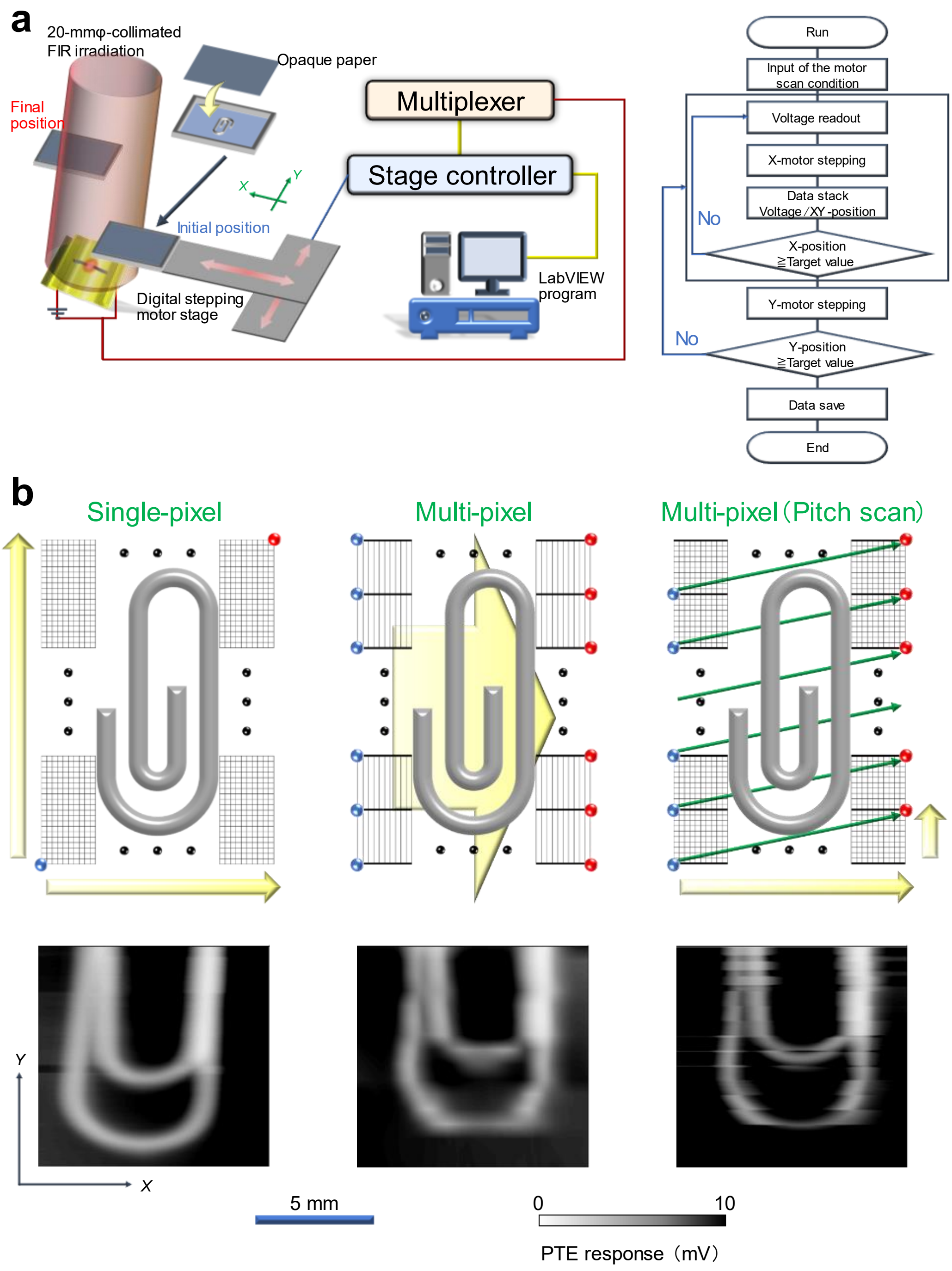

Supplementary Figure 12.

Comparison of photo-thermoelectric (PTE) image resolution based on pixel number.

a, Experimental setup of the transmissive single-pixel XY-scan PTE imaging. **b,** Non-destructive transmissive PTE images (far-infrared (FIR: λ = 10.3 μm)) of concealed metal clip obtained by carbon nanotube (CNT) film photo-imager with different pixel number. The resolution of images obtained by the one-dimensional multi-pixel array scanner can be improved through the simple complementation. The image can be acquired by the one-axis scanning using the multi-pixel array imager. In this scheme, the information between the pixel-pitch areas is missed, resulting in lower image resolution than that obtained by the single-pixel XY scanning. However, combining the aforementioned one-axis scanning and the pixel array shifting between each pixel-pitch enables us to enhance the image resolution. The total imaging time can be reduced to 1/n times between the single-pixel XY scanning and multi-pixel one-axis array scanning with the pixel-pitch shifting, where n corresponds to the pixel number. Here, the configurations of the multi-pixel array imager are as follows: 10

pixels, 1-μm-thickness × 4-mm-length × 0.5-mm-width for each pixel, 0.5 mm for the distance of each pixel. The imaging area is 10 × 10 mm^2 for each measurement. Each X/Y-axis was scanned per every 100 μm for the single-pixel measurement. The X-axis was scanned per every 100 μm, and the array imager was shifted per every 100 μm in the pixel-pitch (Y-axis) for the multi-pixel measurement.

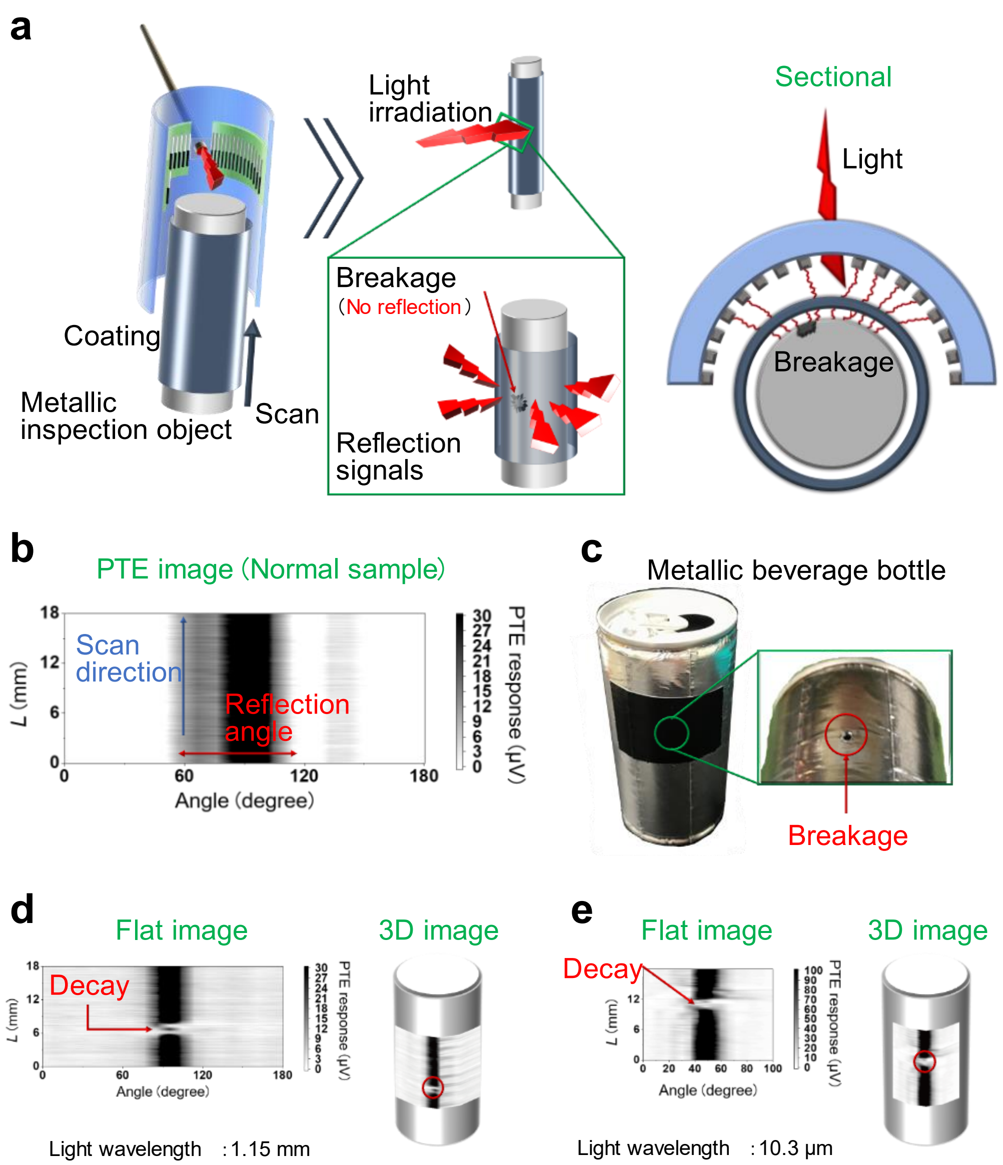

Supplementary Figure 13.

Fundamental operation mechanism of the reflective multi-view three-dimensional (3D) capsule photo-thermoelectric (PTE) imager.

a, Schematic of the non-destructive reflective multi-view stereoscopic capsule photo-monitoring of a defective metallic columnar object. The breakage outer surface of a target column can be identified by detecting locally attenuated reflection signals due to the lack of a reflective surface. **b,** Reflective multi-view capsule photo-monitoring of a normal metallic beverage bottle in the sub-terahertz (sub-THz: $\lambda = 1.15$ mm) frequency region. **c,** Photographs of a metallic beverage bottle where a tiny breakage was concealed under an opaque coating. The breakage was visualised by detecting locally attenuated reflection signals corresponding to its location, via scanning in both **d,** the sub-THz frequency region, and **e,** the far-infrared (FIR: $\lambda = 10.3$ μm) frequency region.

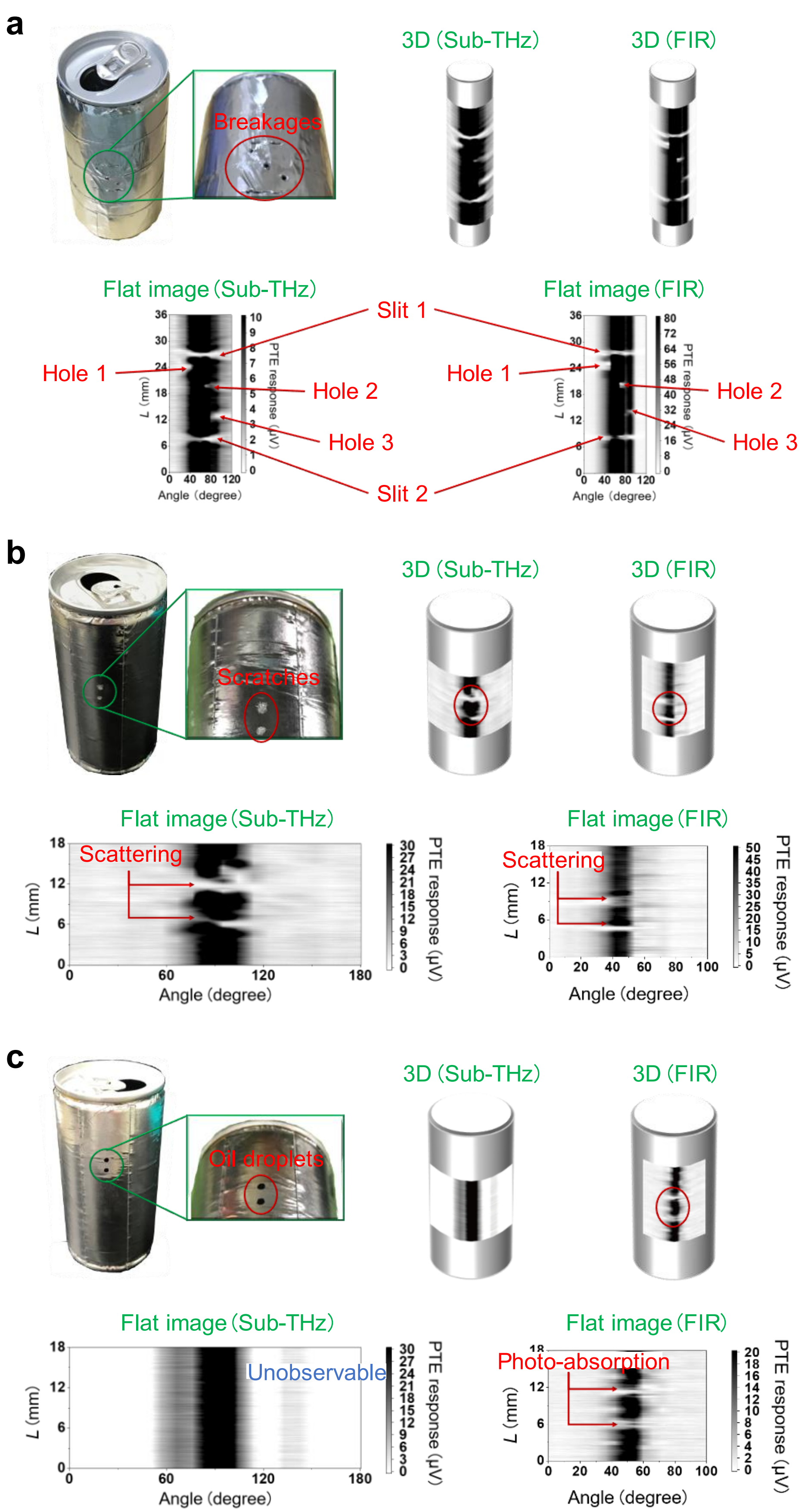

Supplementary Figure 14.

Reflective multi-view three-dimensional (3D) capsule multi-frequency band photo-monitoring between the sub-terahertz (sub-THz: λ = 1.15 mm) and the far-infrared (FIR: λ = 10.3 μm) frequency regions.

a, Non-destructive reflective multi-view stereoscopic capsule photo-monitoring of a metallic bottle where tiny breakages and slits were concealed under an opaque coating. These defects were visualised for both scanning

frequencies by comprehensively detecting the locally attenuated reflection signals, which correspond to their locations. **b,** Non-destructive reflective multi-view stereoscopic capsule photo-monitoring of a metallic bottle with tiny surface scratches concealed under an opaque coating. The scratches were visualised for both scanning frequencies by detecting the locally attenuated reflection signals due to the scattering, which corresponds to their locations. **c,** Non-destructive reflective multi-view stereoscopic capsule photo-monitoring of a metallic bottle where tiny oil droplets were concealed beneath an opaque coating. Although the oil droplets were visualised in the FIR frequency region by detecting locally attenuated reflection signals corresponding to their locations, those were unobservable in the sub-THz frequency region. This phenomenon was attributed to the significant difference between the transmittance of the oil droplets in these scanning frequencies. The corresponding transparency was measured as follows: 51 % for the continuous wave (CW) irradiation (λ = 10.3 μm), 95 % for the CW irradiation (λ = 1.15 mm). Therefore, the locally attenuated reflection signals shown in the obtained FIR image can be derived from the photo-absorption by the oil droplets.

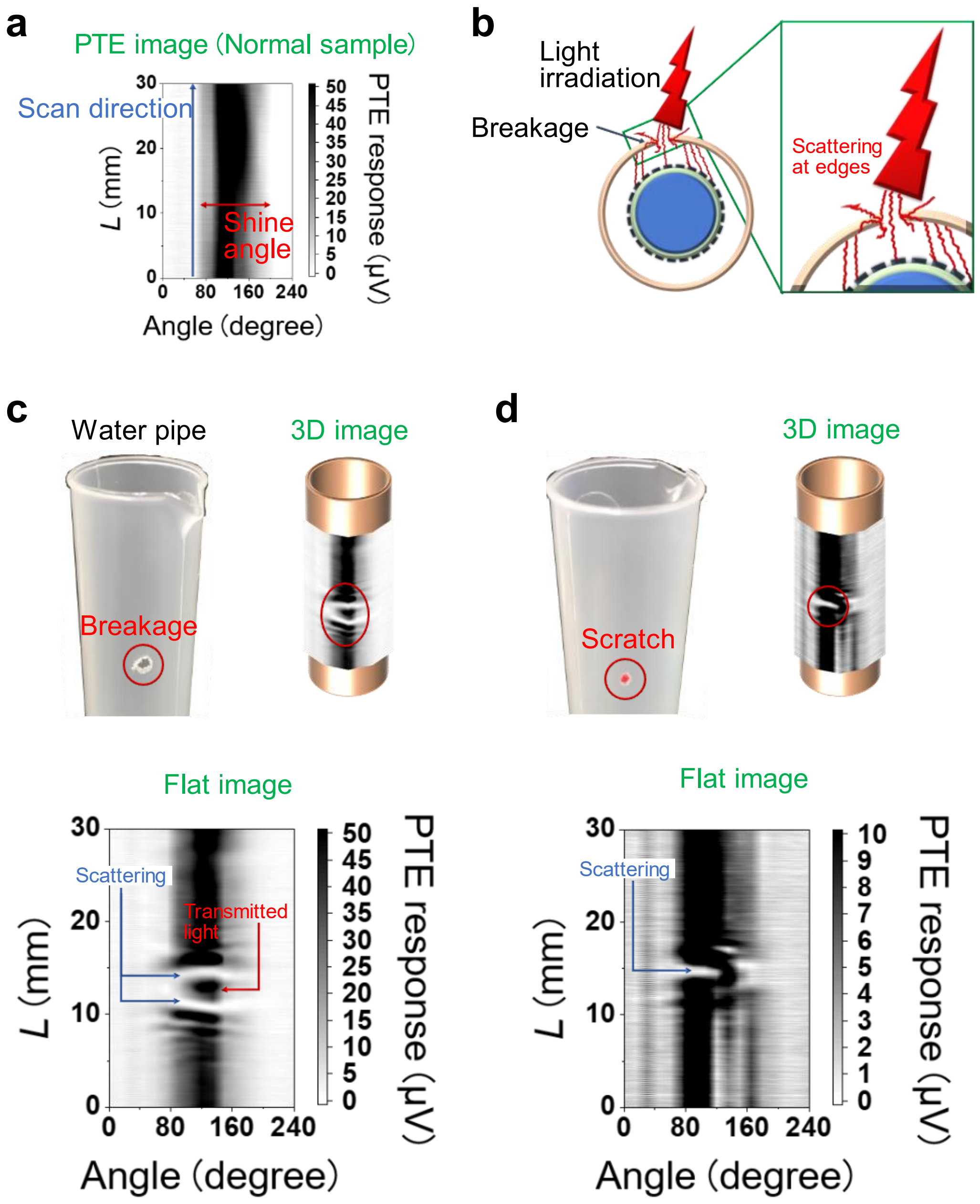

Supplementary Figure 15.

Fundamental operation mechanism of the transmissive multi-view three-dimensional (3D) photo-thermoelectric (PTE) endoscope.

a, Transmissive multi-view endoscopy of a normal plastic water pipe in the sub-terahertz (sub-THz: $\lambda = 1.15$ mm) frequency region. **b,** Cross-sectional schematic of the transmissive multi-view stereoscopic endoscopy of a defective plastic cylindrical object. The breakage on the target cylinder can be identified by detecting locally attenuated transmission signals due to the scattering at the edge of the hole and transmission signals passing through the hollow region. **c,** Non-destructive transmissive multi-view stereoscopic endoscopy of a plastic water pipe (in the sub-THz frequency region) where a tiny breakage was concealed beneath an opaque coating. The breakage was visualised by detecting both locally attenuated transmission signals due to the scattering, which corresponds to the location of hole edges, and the local transmission signals, which correspond to the location of the hole centre. **d,** Non-destructive transmissive multi-view stereoscopic endoscopy of a plastic water pipe (in the sub-THz frequency region) where a tiny surface scratch was concealed under an opaque coating. The scratch was visualised by detecting locally attenuated transmission

signals due to the scattering, which corresponds to the location of the scratch.

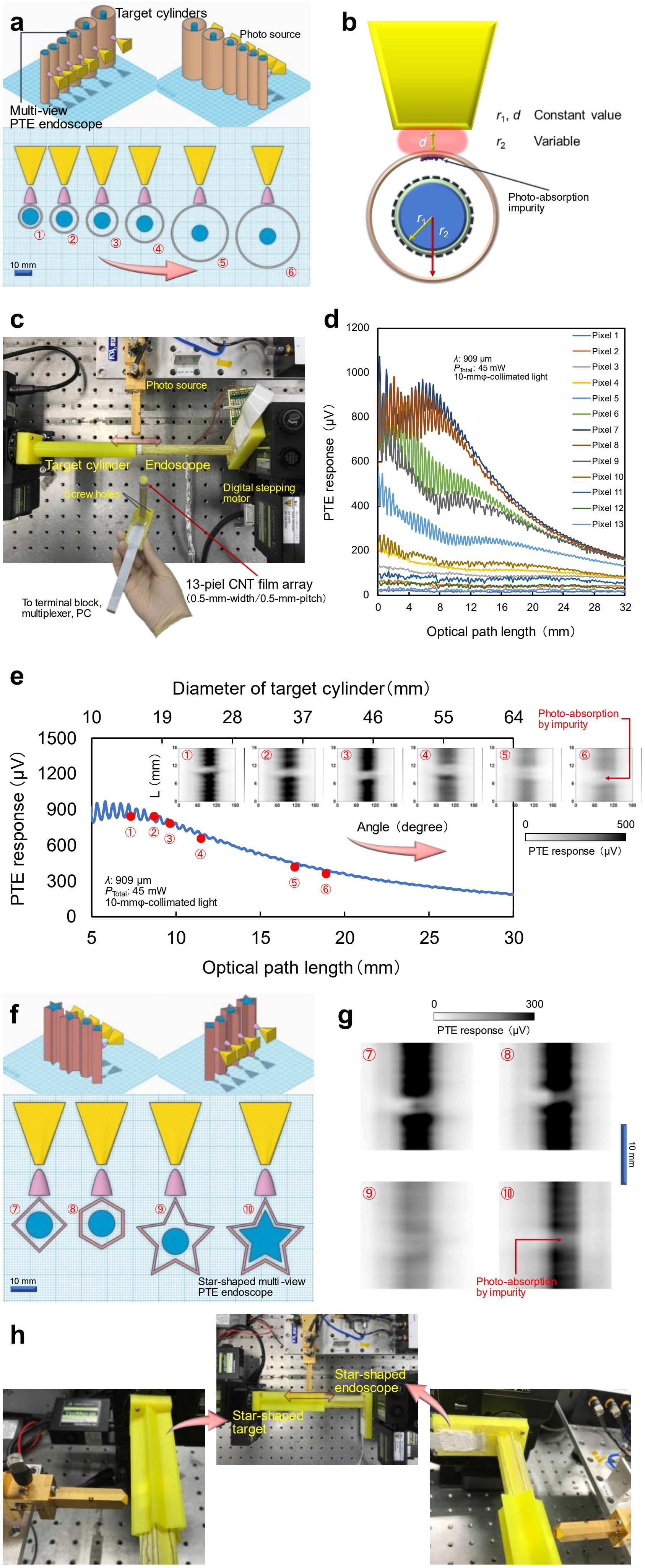
a
Target cylinders
Photo source
Multi-view
PTE endoscope
①
②
③
④
⑤
⑥
10 mm
b
r_1, d Constant value
r_2 Variable
d
Photo-absorption
impurity
r_1
r_2
c
Photo source
Target cylinder
Endoscope
Screw holes
Digital stepping
motor
To terminal block,
multiplexer, PC
13-piel CNT film array
(0.5-mm-width/0.5-mm-pitch)
d
λ: 909 μm
P_{Total}: 45 mW
10-mmφ-collimated light
Pixel 1
Pixel 2
Pixel 3
Pixel 4
Pixel 5
Pixel 6
Pixel 7
Pixel 8
Pixel 9
Pixel 10
Pixel 11
Pixel 12
Pixel 13
PTE response (μV)
Optical path length (mm)
0
200
400
600
800
1000
1200
0
4
8
12
16
20
24
28
32
e
Diameter of target cylinder (mm)
10
19
28
37
46
55
64
Photo-absorption
by impurity
1500
1200
900
600
300
0
PTE response (μV)
L (mm)
Angle (degree)
0
500
PTE response (μV)
λ: 909 μm
P_{Total}: 45 mW
10-mmφ-collimated light
5
10
15
20
25
30
Optical path length (mm)
f
⑦
⑧
⑨
⑩
10 mm
Star-shaped multi-view
PTE endoscope
g
0
300
PTE response (μV)
⑦
⑧
⑨
⑩
10 mm
Photo-absorption
by impurity
h
Star-shaped
endoscope
Star-shaped
target

Supplementary Figure 16.

Imaging performance evaluation of the transmissive multi-view stereoscopic photo-thermoelectric (PTE) endoscope against the size, shape, and structure of target cylinders.

Comparisons of PTE images obtained by the multi-view stereoscopic endoscopy for the different size, shape , and structure of targets were performed to further specify the fundamental performance of the proposed scheme. **a,** Schematic of the prepared circular cylinder targets. Both the targets and supporting column of the endoscope module were fabricated via three-dimensional (3D) printing. The diameters are as follows: 15, 18, 20, 24, 36, and 40 mm for the circular cylinders and 10 mm for the supporting column. The wall-thickness of each target was designed to 1 mm. **b,** Simple diagram of the measurement conditions. While the diameters of the targets are varied, the distance between the sub-terahertz (sub-THz: λ = 909 μm, 10-mmφ-collimated irradiation) photo-source and the targets, and the radius of the endoscope module are maintained in constant values. Therefore, the total optical path length changes depending on the radius of each used circular cylinder targets. The *d* in the figure is 5 mm. Here, a small light-absorbing impurity was concealed inside each cylinder target. **c,** Photographs of the experimental setups. The multi-pixel carbon nanotube (CNT) film PTE image sensor array sheet was firmly attached to the outer surface of the supporting columnar substrate resin. Both the endoscope module and cylinder targets were mounted on the digital stepping motor stages via screw holes, which were also designed by 3D CAD software. **d,** Changes in the PTE responses of the endoscope module vs. the total optical path length. The measurement was conducted without target cylinders. Here, the photo-response noise in the shorter optical path length region of the graph corresponds to the sub-THz interference fringes. **e,** Changes in the PTE images of the endoscope module with different diameter of the circular cylinder targets. Degradation of the obtained PTE images was observed for larger target cylinders following the photo-response attenuation in longer optical path length, as shown in the photo-response – optical path length characteristic of pixel no. 7. However, the concealed photo-absorbing impurity was still identified non-destructively for the cylinder with a diameter of 40 mm, which is four times larger than that of the endoscope module. The impurity was captured by locally detecting attenuated transmission signals on it. **f,** Schematic of the prepared non-circular cylinder targets. Here, each target was fabricated via 3D printing, as well. **g,** Changes in the PTE images of the column-shaped and star-shaped endoscope module with different structure of the targets. The obtained PTE images indicate our endoscope module is also available for the simple non-circular 3D-structured targets, such as a square and hexagon cylinders. Simultaneously, the result infers that the free-form devising of the endoscope module eases the non-destructive inspection of the complicatedly-structured target, including a star-shaped cylinder. For the use of the column-shaped endoscope module against the star-shaped cylinder, the structural mismatch between them resulted in degradation of the PTE image due to the excessive longer optical path length. Therefore, the effective use of 3D printing, where supporting resin of the sensing module is flexibly designed to adjust the target structures, facilitates the operating-environment-independent systemising together with the freely attachable photo-imager devising. **i,** Photographic scheme of the proposed transmissive multi-view stereoscopic star-shaped PTE endoscopy.

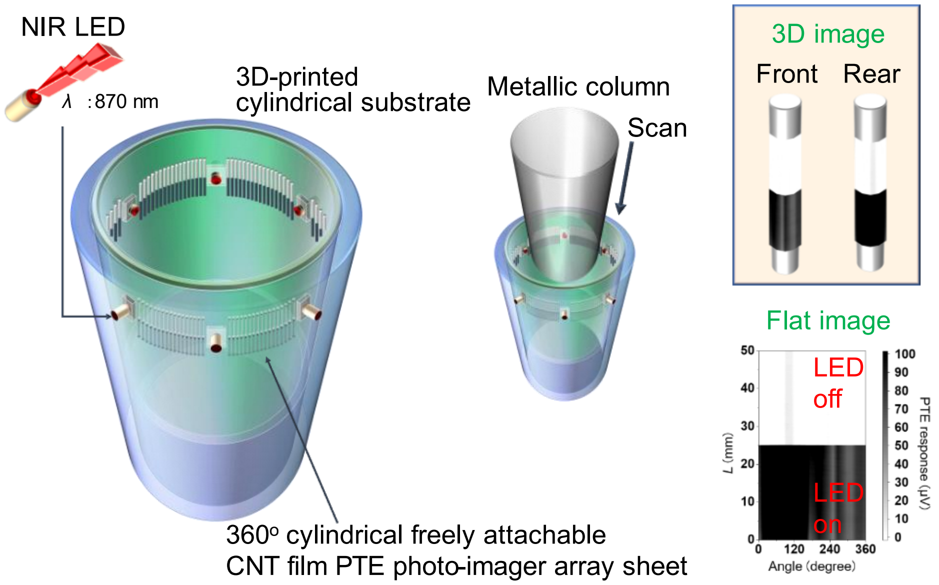

Supplementary Figure 17.

Fundamental operation mechanism of the source-implanted portable 360°-view stereoscopic photo-thermoelectric (PTE) imager with carbon nanotube (CNT) films.

The three-dimensional (3D) image obtained by scanning the metallic column indicates that the uniform behaviour of the reflection signals over a 360° viewing-angle in response to the on/off operations of the implanted near-infrared light emitting diodes (NIR LEDs: $\lambda = 870$ nm).

Supplementary References

1. Suzuki, D., Oda, S., Kawano, Y. A flexible and wearable terahertz scanner, *Nat. Photonics* **10**, 809–813 (2016).
2. Suzuki, D., Ochiai, Y., Kawano, Y. Thermal Device Design for a Carbon Nanotube Terahertz Camera, *ACS Omega* **3**, 3540–3547 (2018).
3. Nonoguchi, Y. et al. Simple Salt‐Coordinated n‐Type Nanocarbon Materials Stable in Air, *Adv. Funct. Mater.* **26**, 3021‐3028 (2016).
4. Cheng, Z. et al. Temperature Dependence of Electrical and Thermal Conduction in Single Silver Nanowire, *Sci. Rep.* **5**, 10718 (2015).
